# Biosorption and Biodegradation of the Environmental Hormone Nonylphenol By Four Marine Microalgae

**DOI:** 10.1038/s41598-019-41808-8

**Published:** 2019-03-27

**Authors:** Luyun Wang, Han Xiao, Ning He, Dong Sun, Shunshan Duan

**Affiliations:** 1 Department of Ecology, Institute of Hydrobiology, School of Life Science and Technology, Jinan University, Guangzhou, P.R. China; 2 College of Life Science and Resources and Environment, Yichun University, Yichun, 336000 Jiangxi China

## Abstract

Microalgae are the most abundant microorganisms in aquatic environments, and many possess the ability to remove organic contaminants. The presence of endocrine disruption compounds (EDCs) in many coastal marine systems and their associated risks have elicited great concern, especially in the case of nonylphenol (NP), which is classified as a priority contaminate by the U.S. EPA. In this context, batch experiments were conducted to investigate the intracellular absorption, extracellular adsorption and biodegradation of NP by four species of marine microalgae: *Phaeocystis globosa*, *Nannochloropsis oculata*, *Dunaliella salina* and *Platymonas subcordiformis*. The results showed a sharp reduction of NP in medium containing the four microalgal species during the first 24 h of incubation, and the four species exhibited the greatest capacity for NP adsorption and absorption within 24 h of culture. However, the amount of NP absorbed and adsorbed by all four microalgae decreased with increasing time in culture, and intracellular absorption was greater than extracellular adsorption. After 120 h of exposure to NP, the four species could biodegrade most of the NP in the medium, with efficiencies ranging from 43.43 to 90.94%. In sum, we found that the four microalgae have high biodegradation percentages and can thus improve the bioremediation of NP-contaminated water.

## Introduction

Alkylphenol polyoxyethylene ethers (APEs) are non-ionic surfactants that are used extensively in various industrial, agricultural and household applications. Many studies have shown that nonylphenol (NP), which is a major degradation product of alkylphenol ethoxylates under natural conditions, presents considerable residual and ecological risk; it is difficult to degrade and transform and is instead adsorbed to solid suspended objects or sediment^1–5^. NP is in the class of typical environmental hormones (EH) known as endocrine disruption compounds (EDCs); it is commonly identified in air, water, sediment and biota and represents a carcinogenic threat to humans^6^. However, persistent organic pollutants can be removed by artificial methods such as photodecomposition, electrochemical and bioactive carbon fibres^7–9^ that have the advantages of high removal and rapid reaction rates, but they may produce secondary pollution and are expensive. Therefore, bioremediation is considered a green and sustainable mitigation option.

Bioremediation technology allows the biological-specific decomposition of wastewater to provide complete degradation at low cost and without energy consumption, among other advantages^10^. In natural near-shore marine systems, microorganisms, plants and algae have the ability to purify water bodies contaminated with organic substances. As primary producers, microalgae play a vital role in aquatic ecosystems, and many species can degrade organic compounds. Microalgae have the advantages of adsorption capacity, a high surface area:volume ratio, wide distribution range, rapid metabolism, low cost, and abundant availability^11–13^. In a water body, microalgae directly contact and interact with pollutants, but some toxic pollutants can inhibit their growth^14^ and affect their physiological ecology^15^. Microalgae can also biodegrade or biotransform organic pollutants via metabolic action, and the mechanisms for removal include accumulation and degradation, which includes both transformation and mineralization^16^. Hendrik studied the degradation of the pesticide endosulfan by blue-green algae and found that the algae exhibited strong biodegradability^17^. Another researcher studied the adsorption, uptake and degradation effects of *Scenedesmus obliquus* on NP and octylphenol (OP) in water and reported that more than 89% of NP and 58% of octylphenol (OP) in the medium were removed by the microalgae after 5 days of incubation, and the highest removal efficiency was close to 100%^18^. In addition, studies of the adsorption, biotransformation and degradation of NP, bisphenol-A (BPA) and other environmental hormones have been reported in the microalgae of the genera *Chlorella*, *Scenedesmus*, and *Fibrea* as well as other species^19^. Microalgae can also absorb and biologically accumulate certain pollutants, which can then be transmitted up the food chain via biological amplification^20^. *Cladophora glomerata* can exert a bio-enrichment effect on NP so that the concentration in the organism is 10,000 times higher than the concentration in the environment^21^, and *Isochrysis galbana* can enrich soil NP at an initial concentration of 100 μg/L by 6490-fold, while 77% of the compound can be adsorbed and absorbed in one hour. In microalgae used as feed, the high enrichment of NP can impact organisms at higher trophic levels such as rotifers and zebrafish^20^. The above findings have led to research on the selection of microalgae species that are highly capable of degrading organic pollutants, and the use of microalgae for the bioremediation of contaminated water has been proposed as a beneficial strategy when implementing environmental bio-refineries^10^. However, there have been limited studies of the application of marine microalgae to degrade NP. The four microalgae examined in this study, *Phaeocystis globosa*, *Nannochloropsis oculata*, *Dunaliella salina* and *Platymonas subcordiformis*, are common and widely distributed species in aquatic ecosystems. In addition, since these four microalgae are rarely used for NP degradation, the data from this study can be used to supplement knowledge related to NP treatment. The purpose of this work was to study (1) the toxic effect of NP on four marine algae—*P. globosa*, *N. oculata*, *D. salina* and *P. subcordiformis*—and to assess whether they are NP-tolerant species; (2) the capability of these four species to remove and biodegrade NP from NP-contaminated aquatic environments; and (3) the mechanisms underlying NP bioaccumulation, biodegradation and removal capability of these four microalgae.

## Results

### Growth of the four microalgae exposed to NP

**Figure 1:**
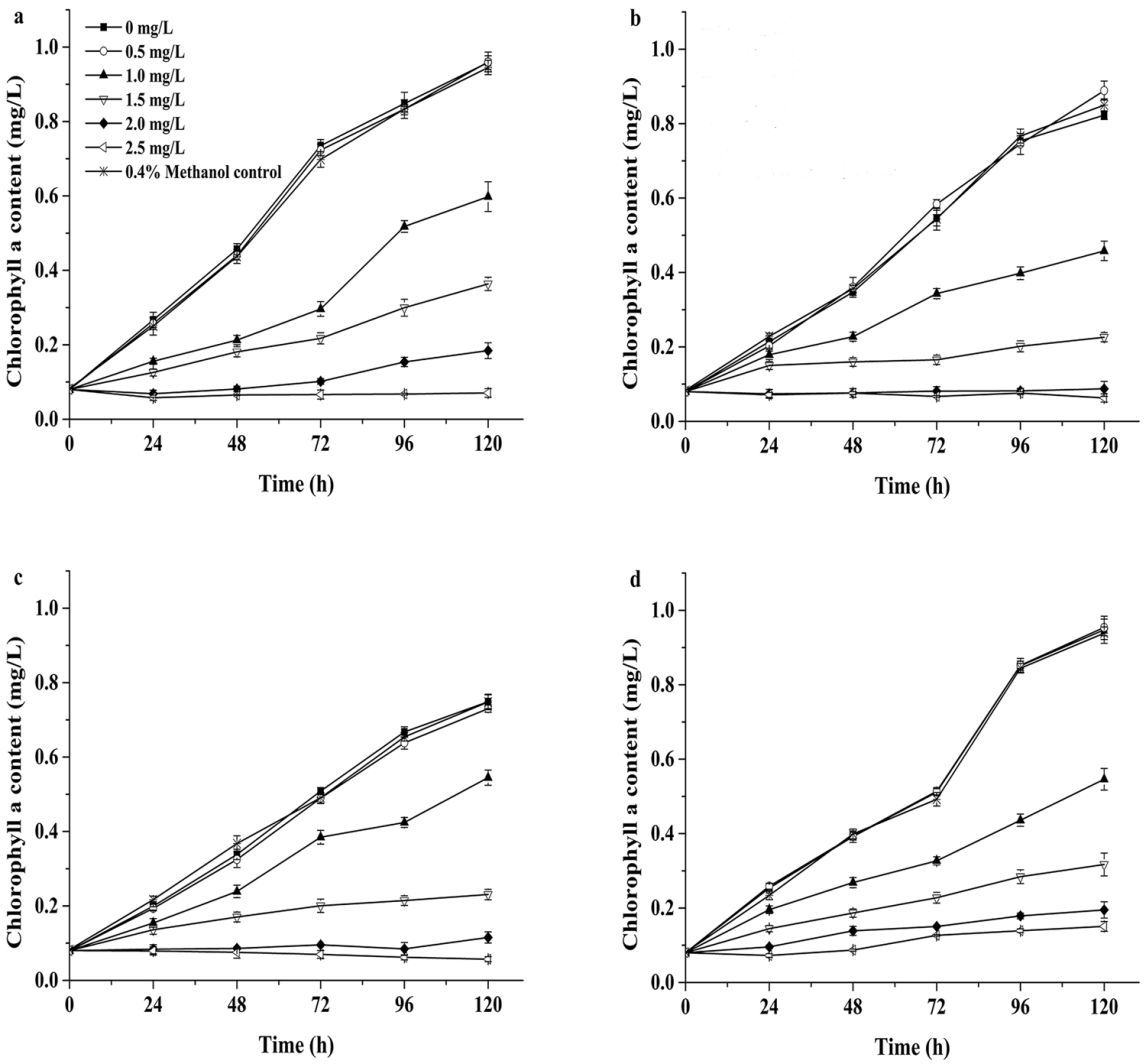
Effect of the NP concentration on the Chla content. (**a**) *P. globosa*, (**b**) *N. oculata*, (**c**) *D. salina* and (**d**) *P. subcordiformis*. The values are the means ± standard deviations (SDs) (*n* = 3).

The growth, according to changes in Chla content, and the effects of NP on the four microalgae showed a significant time-dose effect (Fig. 1). Statistically non-significant inhibition of the Chla concentration was observed at 0.5 mg/L compared to the control (p > 0.05), and a significant inhibition of Chla concentration was observed at 1.0 mg/L compared to the control (p < 0.05). The Chla concentration obviously decreased when NP concentrations were higher than 1.5 mg/L, and the inhibitory effects were significantly enhanced with increasing concentrations of NP after a 24-h exposure phase (Fig. 1). The 96-h EC_50_ of NP for the four microalgae was in the order of *P. subcordiformis* > *P. globosa* > *D. salina* > *N. oculata*. The EC_50_ values for the four microalgae were more than 1.0 mg/L; the EC_50_ of *P. subcordiformis* was the greatest at 1.497 mg/L, and the EC_50_ of *N. oculata* was the lowest at 1.004 mg/L (Table 1). There was no significant difference between *D. salina* and *N. oculat*a under each treatment (*p > *0.05). A linear relationship among algal density (cells/mL), dry weight and Chla content was established (Table S2). The results revealed a positive correlation between cell density and Chla content in the four microalgae and a positive correlation between microalgae biomass and Chla content.

**Table 1:** The 96-h EC_50_ values of NP for the four microalgae.

Microalgal species	Regression equation	R^2^	EC_50_ (mg/L)
*P. globosa*	y = 0.4938 x − 0.1257	0.94933	1.138
*N. oculata*	y = 0.4806 x − 0.0493	0.88678	1.004
*D. salina*	y = 0.5008 x − 0.1065	0.93678	1.076
*P. subcordiformis*	y = 0.3887 x + 0.0543	0.88792	1.497

Noted: x is the Chla concentration, and y is the NP concentration.

### Removal of NP by the four microalgae

**Figure 2:**
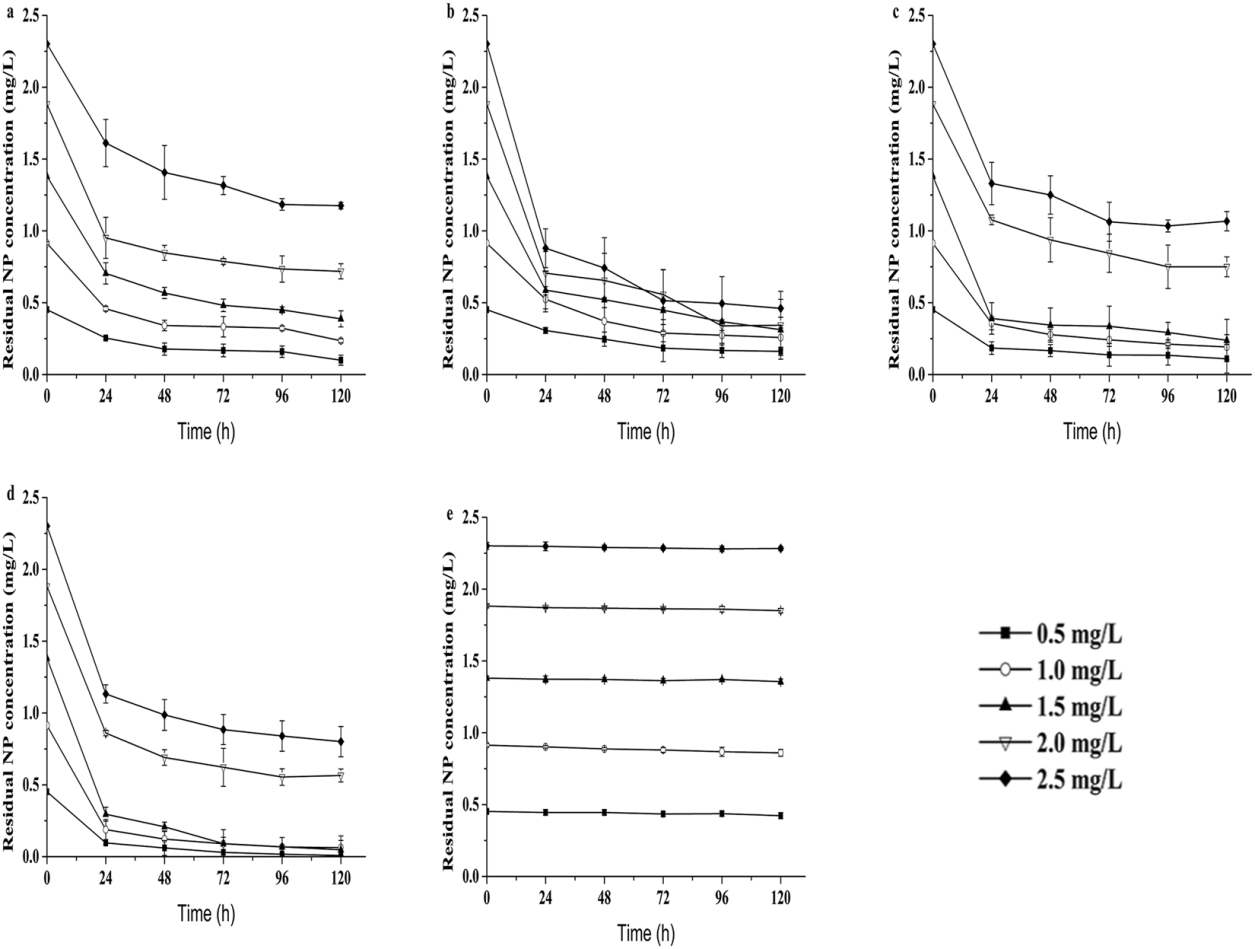
Temporal changes in the residual NP concentrations in the medium of the four algal cultures. (**a**) *P. globosa*, (**b**) *N. oculata*, (**c**) *D. salina*, (**d**) *P. subcordiformis* and (**e**) controls at different initial NP concentrations. Values are the mean ± SD of three replicates.

The concentrations of NP in the incubation culture of the four microalgae is expressed in Fig. 2. The NP concentrations in the medium quickly declined in the treatment with algae. Within 24 h, NP was rapidly removed by the four microalgae, with removal efficiencies of 22.76–78.87% under the five concentration treatments. During the first 24 h, NP was rapidly removed by *P. globosa*, *N. oculata*, *D. salina* and *P. subcordiformis* from 1 mg/L to approximately 441.8 μg/L, 524.2 μg/L, 355.9 μg/L and 188.4 μg/L, respectively, which was equivalent to the removal of 43.14%, 34.01%, 52.43% and 70.75% of the initial amount of NP from the medium. The NP level in the medium decreased further to final concentrations of 235.1 μg/L, 257.4 μg/L, 192 μg/L and 62.3 μg/L NP, respectively, at the end of the 120-h period, and these values accounted for a 74.18%, 72.63%, 79.02% and 92.12% reduction of the initial amount of NP, respectively. After 5 days of culture, the NP removal efficiencies obtained with *P. globosa*, *N. oculata*, *D. salina* and *P. subcordiformis* from initial NP concentrations of 0.5–2.5 mg/L were 66.37%, 74.82%, 69.86%, and 82.38%, respectively. The NP concentrations in the control treatments showed little variation over 120 h, which indicated that photolysis played only a small role in the dissipation of NP.

The reduction of NP in the medium appeared to follow first-order kinetics. Table 2 shows the degradation kinetics equations and parameters of NP removal by the four microalgae. The NP removal statistics can be fitted better to the first-order model with half-lives of 2.188, 2.045, 1.488 and 1.285 days, respectively.

**Table 2:** Kinetic equations for NP (1 mg/L) degradation by the four microalgae.

Microalgal species	Biodegradation kinetic equation	K	t_1/2_	R^2^	*p*
*P. globosa*	C_t_/C_0_ = 0.8927e^−0.3168t^	0.3168	2.188	0.7982	*p < *0.01
*N. oculata*	C_t_/C_0_ = 0.9282e^−0.3388t^	0.3388	2.045	0.8908	*p* < 0.01
*D. salina*	C_t_/C_0_ = 0.9052e^−0.4671t^	0.4671	1.484	0.8023	*p* < 0.01
*P. subcordiformis*	C_t_/C_0_ = 0.8713e^−0.5394t^	0.5394	1.285	0.5867	*p* < 0.01

### NP adsorbed onto the cell surfaces and absorbed into cells

**Figure 3:**
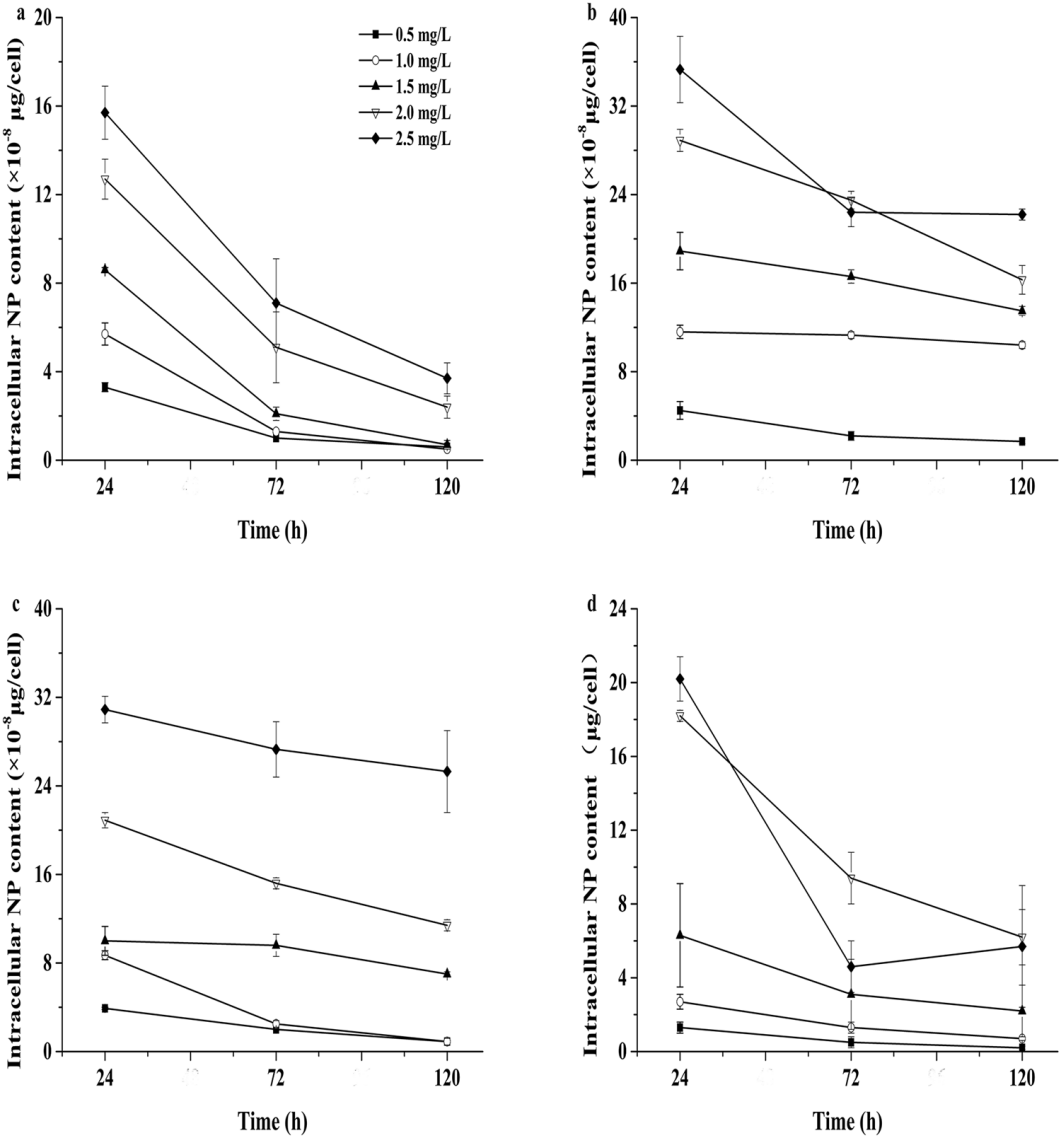
The amounts of intracellular NP measured after 24, 72 and 120 h of treatment in the four microalgae. (**a**) *P. globosa*, (**b**) *N. oculata*, (**c**) *D. salina*, and (**d**) *P. subcordiformis*. Values are the mean ± SD (n = 3).

**Figure 4:**
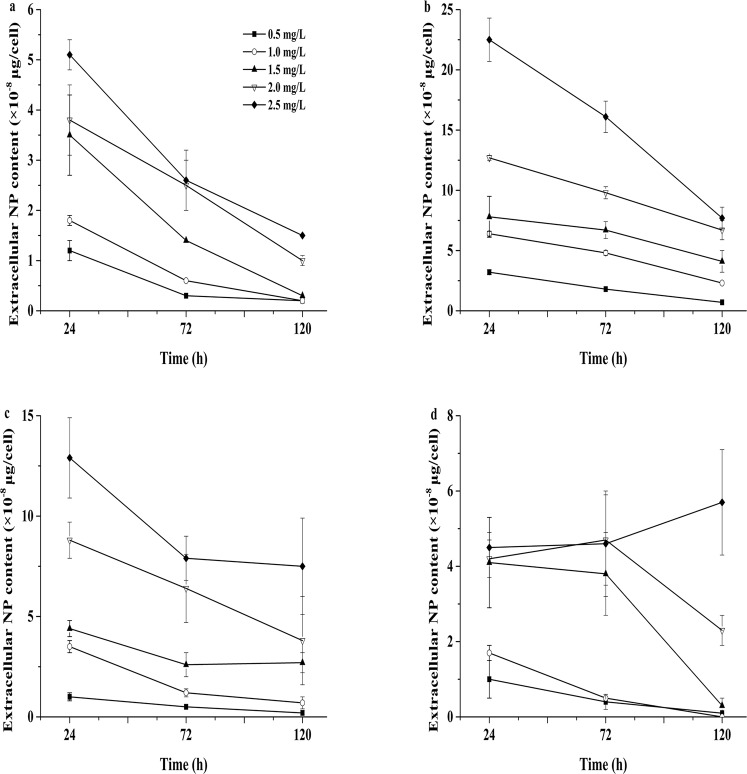
The amounts of extracellular NP measured in the four microalgae after 24, 72 and 120 h of treatment. (**a**) *P. globosa*, (**b**) *N. oculata*, (**c**) *D. salina*, and (**d**) *P. subcordiformis*. Values are the mean ± SD (n = 3).

**Figure 5:**
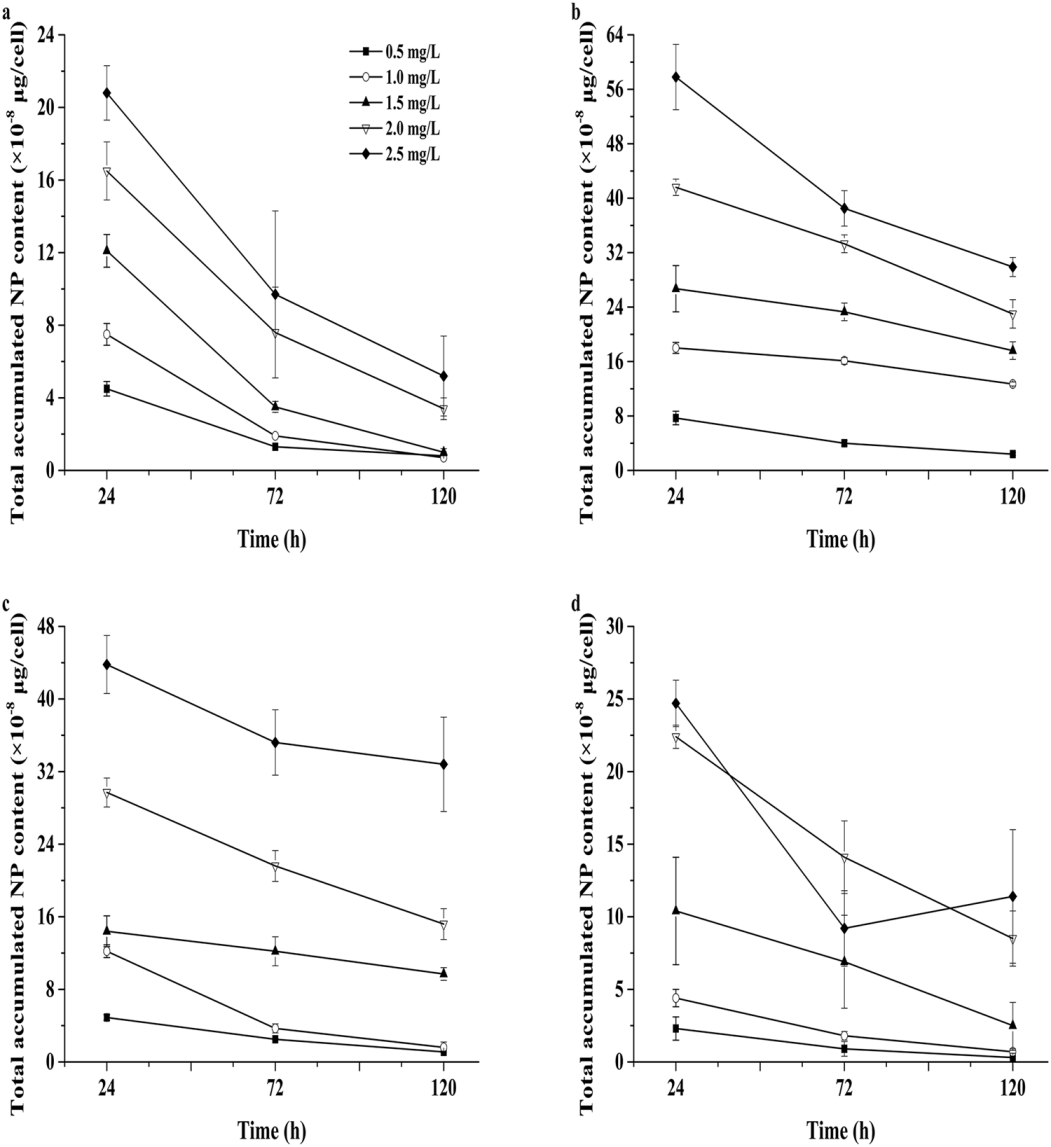
The amounts of total accumulated NP measured in the four microalgae after 24, 72 and 120 h of treatment. (**a**) *P. globosa*, (**b**) *N. oculata*, (**c**) *D. salina*, and (**d**) *P. subcordiformis*. Values are the mean ± SD (n = 3).

NP was found to be adsorbed and absorbed by the four tested microalgae (Figs 3 and 4). The four species adsorbed and absorbed a large amount of NP within one day. The concentration of NP was 2.5 mg/L, and *N. oculata* had the highest intracellular absorption of 35.3 ± 3.0 × 10^−8^ μg/cell. With the increase in culture duration, the amount of NP adsorbed and absorbed by the four microalgae decreased (Figs 3 and 4), and the NP residues in the culture solutions of the four microalgae under different concentrations of NP showed a decreasing trend. The NP concentration was measured in algal cells under the different treatments, and Figs 3 and 4 show that the intracellular NP contents of the four microalgae were higher than those of the extracellular NP contents. The amounts of NP adsorbed and absorbed in algal cells declined with culturing time. The contents of NP accumulated in the cells of the four microalgae were higher in the first 24-h period than those after 120 h of culture (Fig. 5).

### The NP biodegradation, removal and biosorption ratios of the different species of microalgae

**Figure 6:**
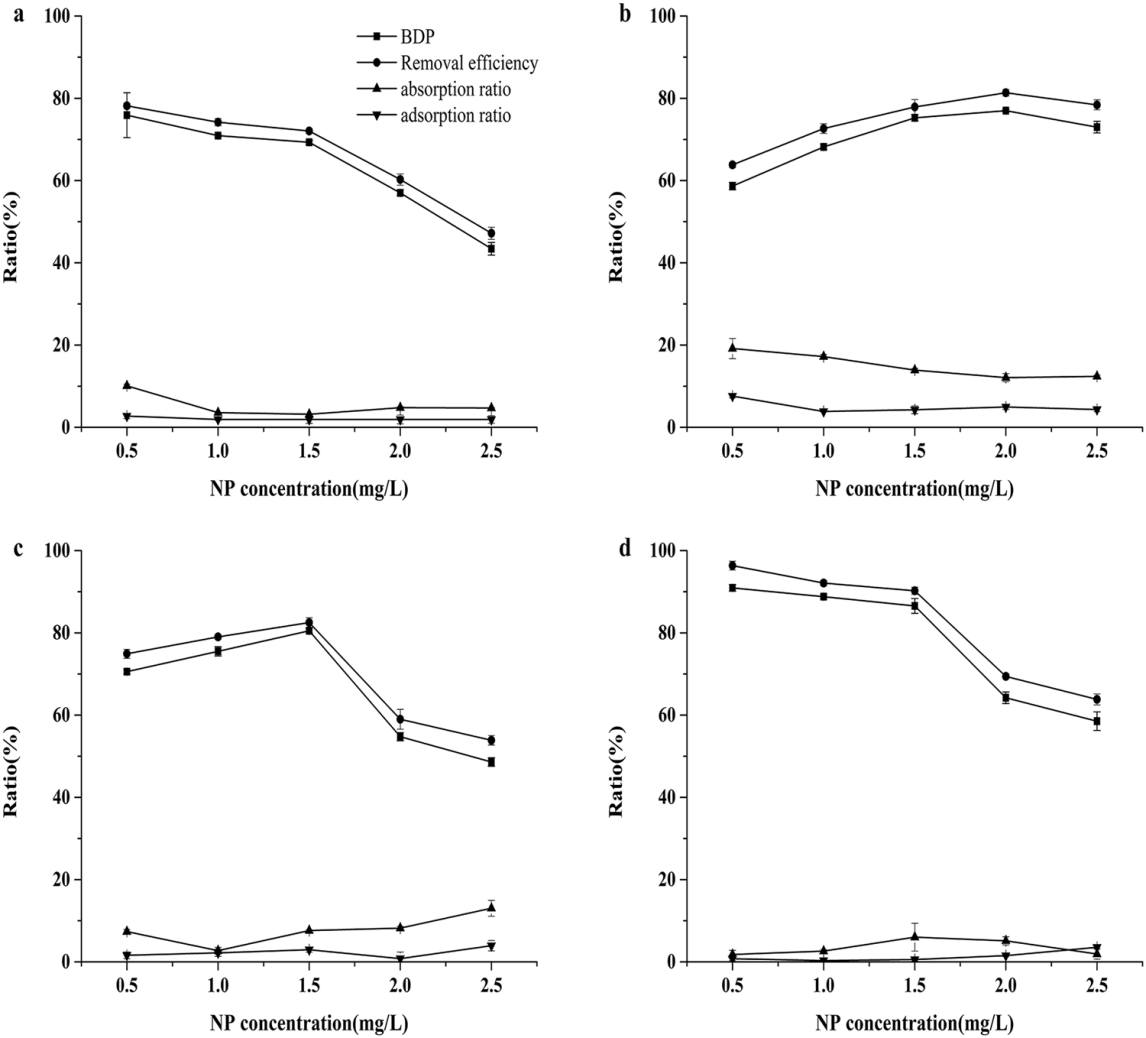
The NP biodegradation, removal and biosorption ratios of the four species of microalgae. (**a**) *P. globosa*, (**b**) *N. oculata*, (**c**) *D. salina*, and (**d**) *P. subcordiformis* at the end of 120 h. (Values show the mean ± SD for three replicates.) Means followed by different concentrations are significantly different according to the LSD test at *P* < *0.05*.

The NP biodegradation percentage changed with the NP concentration. As seen in Fig. 6d, *P. subcordiformis* had a strong biodegradation effect at low concentrations (0.5 mg/L), and the biodegradability of NP was significantly reduced with increasing NP concentrations (*p* < 0.05). *P. globosa* and *P. subcordiformis* exhibited the same biodegradation trend of a stronger biodegradation effect at low NP concentrations than at high NP concentrations (Fig. 6), and *P. subcordiformis* acclimated better than *P. globosa*. The *N. oculata* biodegradation ratio at low NP concentrations was significantly lower than that at high concentrations (*p* < 0.05). When the concentration of NP was 0.5–1.5 mg/L, the *D. salina* biodegradation percentage increased as the concentration increased, but when the NP concentration was greater than 2.0 mg/L, the *D. salina* NP biodegradation percentage significantly decreased (*p* < 0.05). The biosorption ratios of the four microalgae were higher in the first 24-h period than those after 120 h of culture. The removal of NP by the four species of microalgae was largely due to biodegradation or biotransformation by the algal cells rather than simple adsorption and absorption in the cells (Fig. 6).

## Discussion

The present study showed that the growth of the four marine microalgae showed differences after exposure to NP. At NP concentrations greater than 1.5 mg/L, the inhibitory effects measured after 24 h of culture were enhanced with increases in the NP concentration, and this finding agrees with the results of other studies on *Microcystis aeruginosa*, *Chlorella* species, *Scenedesmus quadricauda*, *Ankistrodesmus acicularis* and *Chroococcus minutus* exposed to NP^22–25^ and of *Stephanodiscus hantzschii*, *Chlorella fusca* and *Monoraphidium braunii* exposed to BPA^26–28^. The lowest Chla content was observed at higher concentrations of NP. A previous study showed that NP treatments induce the overproduction of reactive oxygen species (ROS) to cause oxidant damage, which might be one of the basic reasons for the inhibition of algal growth^29^. Higher concentrations of NP caused peroxidation of chloroplast membranes or increased production of ROS^29,30^. The 96-h EC_50_ value of NP was greater than 1 mg/L for all four microalgae species. For *P. subcordiformis*, a more tolerant species, the 96-h EC_50_ of NP was 1.497 mg/L, whereas *P. subcordiformis* exhibited higher NP biodegradation and biotransformation capabilities but lower adsorption and absorption in algal cells compared with those of the other three species. Torres^31^ suggested that biotransformation can improve the elimination, decontamination and redistribution of contaminants within an organism. Thus, we speculated that the strong tolerance of *P. subcordiformis* to NP might be due to its better acclimation, which allowed its photosynthetic activity to recover from the damage caused by NP.

We found that the residual NP concentrations in the medium after a 24-h exposure phase were clearly lower than the concentrations at the start of the experiment. The amounts of NP that accumulated in the cells of the four microalgae were higher in the first 24-h period than they were after 120 h of culture. We concluded that NP could be quickly removed from the medium and be biodegraded or biotransformed by the four microalgae. *Stephanodiscus hantzschii* had a high removal ability at low BPA concentrations, as BPA was bioaccumulated and biodegraded by the algal cells^26^. When BPA was added to the cultures of eight species of freshwater microalgae, a decrease in the concentration of BPA in the medium was observed in all the cultures^32^. Mafalda S. Baptista found that OP removal obviously increased in the presence of cyanobacteria, with a decrease in the half-life of the compound from 15 days in the absence to 9 days in the presence of cells^33^. In this study, the four microalgal species could adsorb, absorb and biodegrade NP. We determined that the removal of NP from the medium was largely due to biodegradation and biotransformation by the algae because the biodegradation ratio ranged from 43.43% to 90.94%. The capabilities of the four microalgae to biodegrade NP were higher than those of *Cyclotella caspia*, *C. minutus* and *A. acicularis*^25,34^. Differences in biodegradability can be due to the mechanism by which algae accumulate organic pollutants, which is analogous to the partitioning of organic compounds in lipid-water systems^35^. The bioaccumulation capability of algae is related to the lipid content in algal cells, which depends on the growth conditions^36^ and distribution in the cell.

Previous studies have identified the algal growth rate as a vital factor influencing the degradation of target compounds by algae^37^. A higher growth rate of microalgae leads to a higher algal biomass that can reduce the concentration of NP dispersed in the algal cells, thus reducing the NP-induced stress on the microalgae. Moreover, the higher algal biomass can enhance the NP biodegradation or biotransformation ability. We also found that the NP removal efficiency increased with the algal growth rate (Table S1 in the Supplementary Material). However, *N. oculata* displayed the highest biodegradation percentage and removal efficiency under the 2.0 and 2.5 mg/L NP treatments, which could have been due to the high adsorption and absorption in cells. Meanwhile, the higher adsorption and absorption by *N. oculata* with increasing NP indicates that the species could not recover from the NP-induced damage. Thus, *N. oculata* did not show significant growth under NP concentrations of 2.0 and 2.5 mg/L but showed the highest biodegradation percentage and removal efficiency. The biodegradation by the four microalgal species, especially *P. subcordiformis*, was mainly related to the microalgal growth rate. The *P. subcordiformis* has the advantages of strong NP tolerance, an efficient growth rate and high biodegradability. These advantages can be applied in the further study of the bioremediation of NP-contaminated water, and the species may be a good choice for the treatment of environmental hormones.

With initial NP concentrations lower than 0.5 mg/L, the normal and long-term growth of the microalgae are not problematic, but NP concentration that exceed 0.5 mg/L inhibit microalgal growth. At present, the actual concentration of NP in water bodies is less than 0.5 mg/L. Therefore, the four tested microalgae can be used as a component of the natural remediation system to achieve the dual purposes of NP pollution control and biomass culture. The addition of certain density of these microalgae to a bioreactor for the treatment of sewage would achieve the goal of removing NP; however, if the concentration of NP in the sewage is high, the growth of the microalgae could be inhibited and even prevented. The analysis of the degradation products revealed that different organisms utilize different pathways and mechanisms to degrade NP, and the obtained products show differences^38–40^. Therefore, our future research might focus on the NP biodegradation pathways used by different algae and their degradation products.

## Materials and Methods

### Microalgal species and culture conditions

*P. globosa*, *N. oculata*, *D. salina* and *P. subcordiformis* were obtained from the Hydrobiology Research Centre, Jinan University, Guangzhou, China. The four marine microalgae used in this study were axenically cultured following the method described by Su^41^; the growth medium was artificial seawater enriched with f/2 enrichment solution for microalgal cultures. The salinity of the artificial seawater was 30‰, and the primary pH of the medium was 6.5–7.0. The flasks were continuously shaken at 100 rpm. Throughout the experiments, f/2 medium^42^ was used, and the four microalgae were cultured in 2-L Erlenmeyer flasks containing 1 L of medium. The culture was illuminated with a cool, fluorescent light at an intensity of 150 μmol/m^2^·s at the surface of the medium (measured by a LI-250 Light meter, LI-COR, Inc., US) with a 12:12-h light:dark cycle in an environmental chamber at 23 ± 2 °C. The culture was aerated by a mechanical air pump with 0.2 μm filtered air at a rate of 35 mL/min, and it was maintained in an exponential growth phase through repeated sub-culturing with fresh medium every four days. Before the experiment, the microalgal cells were collected by centrifugation at 5000 g and 25 °C for 10 min at the exponential growth phase (around the mid-logarithmic phase), and the cell pellets were then washed twice with double-distilled, sterilized water.

### NP treatments

NP obtained from Sigma-Aldrich (St. Louis, MO, USA) was dissolved in methanol as the stock solution at a concentration of 2000 mg/L. The stock solution was spiked into the culture at different initial NP concentrations: 0.5, 1.0, 1.5, 2.0 and 2.5 mg/L. Methanol at a concentration of 0.4% (v/v) was added to the culture (Fig. S1 in Supplementary Material) in each NP treatment to ensure that the spiked NP was fully dissolved in the medium. Each treatment, as well as the control, was performed in triplicate. The primary concentration of chlorophyll a (Chla) was maintained at 0.08 mg/L in a 250-mL Erlenmeyer flask by resuspending the appropriate amount of microalgal cell pellets in 100 mL of culture. Then, the cultures were incubated at 23 ± 2 °C on a rotary shaker at 100 rpm, and the cultures were illuminated with fluorescent lights at a light intensity of 150 μmol/m^2^·s under a 12:12-h light:dark cycle for 120 h.

### Effects of NP on the growth of the four microalgae

#### Measurement of chlorophyll a content

The algal growth was measured by the daily changes in Chla content. First, 5 mL of microalgae culture was collected by centrifugation at 3000 g for 10 min. The supernatant was discarded, and the pellet was resuspended in 5 mL of extract (acetone: ethanol = 1:1), stored in a 4 °C refrigerator, and centrifuged at 3000 g for 10 min after 24 h. The absorbance of the supernatant was detected at wavelengths of 645 nm and 663 nm with an Agilent 2450 UV–visible spectrophotometer, and the Chla concentration of the extract was calculated using the following formula described by Dai^43^:

$${\rm{Chla}}(\mathrm{mg}/L)=12.7O{D}_{663}-2.69{{\rm{OD}}}_{645}$$

The Chla concentrations of the four microalgae were determined daily. The growth inhibition rates at different NP concentrations were calculated according to the following equation:

$$I=\frac{\mu c-\mu t}{\mu c}\times 100 \% $$

where *I* is the growth inhibition rate, *μc* is the algal growth rate of the control group, and *μt* is the algal growth rate at time *t*. Based on the NP concentration and inhibition rate, the 96-h EC_50_ was then calculated through the linear regression method.

#### Determination of the cell density and dry weight of the four microalgae

A haemocytometer (Marienfeld, Lauda-Königshofen, Germany) was used to determine the cell density. To measure the algal cell dry weight, a 20-mL aliquot of culture was sampled and then filtered through a pre-weighed 0.45-mm-pore Whatman GF/F glass-fibre filter. The filter with algal cells was dried overnight in an oven (101A–E, Shanghai Anting Scientific Instrument Co., Ltd.) at 60 °C until a constant weight was reached. The dry weight of the algal cells was the difference between the final weight after filtration and the initial weight before filtration.

### Experimental determination of the biodegradation of NP by the four microalgae

#### Residual NP concentration in the medium

The concentrations of NP in the culture samples were examined at time intervals of 24, 48, 72, 96 and 120 h. First, 5 mL of medium was sampled from each flask, and the algal cultures were centrifuged at 5000 g for 15 min at 4 °C. The supernatant was extracted with dispersive liquid–liquid microextraction (DLLME), as described by Rezaee *et al*.^44^ with modifications reported by Luo *et al*.^45^. Briefly, 0.2 mL of the chlorobenzene and acetone mixture (1:2) was added to the sample in a 10-mL glass test tube with a conical bottom and screw cap. After gentle mixing, the glass tube was filled with a cloudy, milky solution composed of water/chlorobenzene. Then, the sample was centrifuged at 4500 g for 5 min, and the dispersed fine particles in the extraction phase that settled in the bottom of the tube were removed with a 50-μL microsyringe (zero dead volume, cone tip needle). This extraction procedure was repeated three times, and the sediment fractions were mixed together for further analysis by high-performance liquid chromatography (HPLC) (Agilent, Santa Clara, CA, USA). The entire extraction procedure was carried out at ambient temperature (23 ± 2 °C)^44,45^. The recovery rate of NP from the water was 90–94%.

#### Analysis of NP adsorption onto cell surfaces and absorption into cells

To determine the amount of NP adsorbed onto the surfaces of algal cells, the algal cell pellets from the above section were rinsed with 5 mL of 10% methanol and shaken for approximately 60 s. The NP present in the medium was viewed as surface-adsorbed NP^46^ and was extracted with DLLME following the steps described in section 2.4.1, then analysed via HPLC.

To determine the NP absorbed into the cells, the cell pellets obtained using the above-described procedure were added to an appropriate amount of anhydrous Na_2_SO_4_, sonicated (50% power, ice-bath) for 20 min and extracted with 3 mL of dichloromethane–methanol (1:2 v/v). The extract was centrifuged twice for 5 min at 3500 g, and the solvent fractions were ultimately mixed for further analysis^20^. The results regarding “NP adsorbed onto cell surfaces” and “NP absorbed into cells” were compared to those obtained for a control group without NP^20^.

According to the determined NP concentrations, the NP removal efficiency (R) and biodegradation percentage (BDP) of the algal biomass were calculated as previously described^47^, with minor modifications according to the following equations:

$${\rm{R}}( \% )=100\times ({{\rm{C}}}_{{\rm{i}}}-{{\rm{C}}}_{{\rm{f}}})/{{\rm{C}}}_{{\rm{i}}}$$

where R is the dissolved NP removal efficiency (%) and Ci and Cf are the initial and ultimate concentrations (mg/L) of NP in the solution, respectively. Then,

$$\mathrm{BDP}\,( \% )=100\times ({\rm{Ci}}-{\rm{Cr}}-{\rm{Ca}}-{\rm{Cd}}\times {\rm{Wa}}-{\rm{Cb}}\times {\rm{Wa}})/\mathrm{Ci}$$

where Ci is the primary concentration (mg/L) of NP in the solution, Cr is the remaining concentration (mg/L) in the solution, Ca is the concentration of the abiotic removal (mg/L), Cd is the dry weight concentration (mg/L) of NP adsorbed on the surface of the cell walls, Cb is the concentration (mg/g dry weight) of NP absorbed in algal cells, and Wa is the dry weight of the algal biomass in g/L.

The intracellular NP content (μg/cell) and the extracellular NP content (μg/cell) were calculated according to the following equations:

$${\rm{Intracellular}}\,{\rm{NP}}\,{\rm{content}}=\mathrm{Cb}/\mathrm{cell}\,{\rm{density}}$$

$${\rm{Extracellular}}\,{\rm{NP}}\,{\rm{content}}=\mathrm{Cd}/\mathrm{cell}\,{\rm{density}}$$

where Cb is the dry weight concentration (mg/L) of NP absorbed in the cell and Cd is the dry weight concentration (mg/L) of NP adsorbed on the cell walls.

#### Kinetic equations for NP degradation by the four microalgae

First-order kinetic models can be used to describe algal degradation and are mainly used to describe degradation over time. The kinetic equation for algal degradation of NP can be expressed as follows:

$${{\rm{C}}}_{{\rm{t}}}={{\rm{C}}}_{0}{{\rm{e}}}^{-{\rm{ket}}}$$

where k is the kinetic constant in the first-order reaction, C_t_ is the NP concentration at time t, and C_0_ is the initial NP concentration. Parameter K was obtained through a linear regression between the removal rate (C_t_/C_0_) and the treatment time (t).

#### Determination of NP

NP concentrations were analysed via an Agilent 1100 series high-performance liquid chromatograph (Agilent, Santa Clara, CA, USA) with a fluorescence detector. Each extracted sample was dried with N_2_ gas before the HPLC analysis. The elution was carried out with acetonitrile and Milli-Q water (80:20 v/v) as the mobile phase under isocratic conditions. An XDB-C18 RS column (4.6 × 250 mm, 5 μm) was used. The injection volume was 50 μL, and the flow rate was set as 1 mL/min. The fluorescence detector was set at an excitation wavelength of 230 nm and an emission wavelength of 305 nm. The retention time was 18 min, and the quantification limit for NP was 5 μg/L. The results were compared to those obtained with a control without NP^20^.

#### Statistical analysis

Each experiment was conducted in triplicate, and the mean values and standard deviations (SD) were calculated from the different replicates (n = 3). Statistical analysis was performed using the SPSS 16.0 package (SPSS Inc., Chicago, IL, USA). One-way ANOVA was followed by LSD multiple comparisons and the Wilcoxon test when nonparametric tests were necessary (*p* < 0.05 was considered significant, and *p* < 0.01 was considered highly significant).

## Conclusions

We studied the influence of NP on the growth of the marine microalgae *P. globosa*, *N. oculata*, *D. salina and P. subcordiformis* and evaluated their ability to biodegrade NP in culture. *P. subcordiformis* has a stronger tolerance for NP than do the other three species. Moreover, the four microalgae could remove NP from the medium via biosorption, biodegradation or biotransformation. We conclude that *P. subcordiformis* is an excellent candidate species for the bioremediation of NP-polluted aquatic ecosystems.

## Supplementary information

Figure S1, Table S1 and S2
