# Supplementary material for: Biosorption and Biodegradation of the Environmental Hormone Nonylphenol By Four Marine Microalgae
Source: Sci Rep. 2019 Mar 27;9:5277. doi: 10.1038/s41598-019-41808-8 (PMC6437302; doi:10.1038/s41598-019-41808-8)
Supplement: Supplementary file 1 — Figure S1, Table S1 and S2 [file 41598_2019_41808_MOESM1_ESM.docx]

**Biosorption and biodegradation of the environmental hormone nonylphenol by four marine microalgae**

Luyun Wang^1^, Han Xiao^1^, Ning He^2^, Dong Sun^1^*, Shunshan Duan^1^*

^1^ Department of Ecology, Institute of Hydrobiology, School of Life Science and Technology, Jinan University, Guangzhou, PR China.

^2^ College of Life Science and Resources and Environment, Yichun University, Yichun 336000, Jiangxi, China

***** Corresponding authors: tssduan@jnu.edu.cn, jnu_sundong@163.com.

**Figure S1.**

**
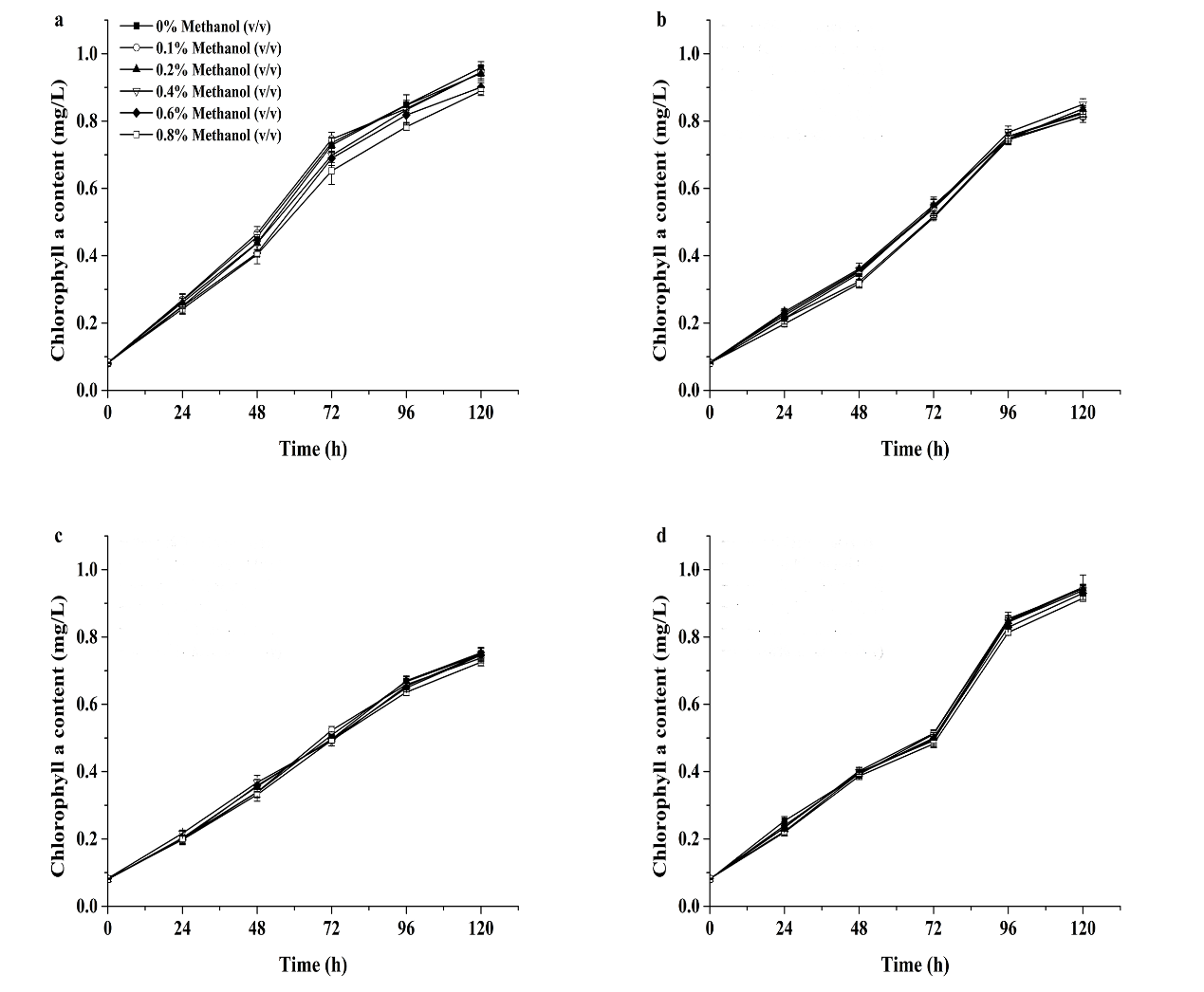
**

**Figure S1. Effect of methanol concentration on the Chla content of (a) *P. globosa*, (b) *N. oculata*, (c) *D. salina* and (d) *P. subcordiformis*.**

**Table s1**. Linear regression equation for the algal growth rate and the removal rate of NP.

| Algal species | Regression eqution (growth rate[X], removal rate[Y]) | R^2^ |
| --- | --- | --- |
| *P. globosa* | Y = 134.17 X - 39.35 | 0.90384 |
| *N. oculata* | Y = 62.385 X – 6.5806 | 0.96645 |
| *D. salina* | Y = 157.07 X – 48.364 | 0.88491 |
| *P. subcordiformis* | Y = 118.15 X – 26.699 | 0.99822 |

**Table s2.** Linear regression equations for cell density and Chla content and dry weight and Chla content

| **Microalgae species** | **Regression equation** | **R^2^** |
| --- | --- | --- |
| *P. globosa* | Cell density (10^4^cells/mL) = 727.16 × Chla content - 20.967 | 0.87197 |
| *N. oculata* | Cell density (10^4^cells/mL) = 650.68 × Chla content - 25.243 | 0.9325 |
| *D. salina* | Cell density (10^4^cells/mL) = 633.08 × Chla content + 11.482 | 0.98183 |
| *P. subcordiformis* | Cell density (10^4^cells/mL) = 701.53 × Chla content -50.942 | 0.909 |
| *P. globosa* | Dry weight (g/L) = 0.0266 × Chla content + 0.0151 | 0.98105 |
| *N. oculata* | Dry weight (g/L) = 0.0294 × Chla content + 0.0159 | 0.94092 |
| *D. salina* | Dry weight (g/L) = 0.0279 × Chla content + 0.0174 | 0.98144 |
| *P. subcordiformis* | Dry weight (g/L) = 0.0429 × Chla content + 0.0165 | 0.92625 |
